# Influence of oxalate ligand functionalization on Co/ZSM-5 activity in Fischer Tropsch synthesis and hydrodeoxygenation of oleic acid into hydrocarbon fuels

**DOI:** 10.1038/s41598-017-09706-z

**Published:** 2017-08-30

**Authors:** Olumide Bolarinwa Ayodele

**Affiliations:** Department of Chemical Engineering, Universiti Teknologi Petronas, 32610 Bandar Seri Iskandar Perak, Malaysia

## Abstract

Achieving high degree of active metal dispersions at the highest possible metal loading and high reducibility of the metal remains a challenge in Fischer Tropsch synthesis (FTS) ﻿as well as in hydrogeoxygenation (HDO)﻿.﻿This study therefore reports the influence of oxalic acid (OxA) functionalization on the﻿ metal dispersion, reducibility and﻿ activity of Co supported ZSM-5 catalyst in FTS and HDO of oleic acid into paraffin biofuel. The Brunauer–Emmett–Teller (BET) results showed that cobalt oxalate supported ZSM-5 catalyst (CoOx/ZSM-5) synthesized from the incorporation of freshly prepared cobalt oxalate complex into ZSM-5 displayed increase in surface area, pore volume and average pore size while the nonfunctionalized cobalt supported on ZSM-5 (Co/ZSM-5) catalyst showed reduction in those properties. Furthermore, both XRD and XPS confirmed the presence of Co° formed from the decomposition of CoOx during calcination of CoOx/ZSM-5 under inert atmosphere. The HRTEM showed that Co species average particle sizes were smaller in CoOx/ZSM-5 than in Co/ZSM-5, and in addition, CoOx/ZSM-5 shows a clear higher degree of active metal dispersion. The FTS result showed that at CO conversion over Co/ZSM-5 and CoOx/ZSM-5 catalysts were 74.28% and 94.23% and their selectivity to C_5+_ HC production were 63.15% and 75.4%, respectively at 4 h TOS. The HDO result also showed that the CoOx/ZSM-5 has higher OA conversion of 92% compared to 59% over Co/ZSM-5. In addition CoOx/ZSM-5 showed higher HDO and isomerization activities compared to Co/ZSM-5.

## Introduction

The depletion of fossil fuel reserves coupled with the attendant environmental pollution from its combustion as well as the recent fall in the crude oil prices globally have generated intensified research into alternative source of energy especially for the transport sector^1, 2^. In all the research areas, the search for sustainable feed stocks and processing techniques seems to be at the fore front^1, 3^. A sustainable feed stock should be renewable and not conflict with the source of food for the populace especially for the low income dwellers in the rural areas of third and developing countries. On the other hand, a sustainable processing technique should maximize profit, minimize waste and have a high turnover and throughput. The two categories of sustainable and renewable energy sources (SRES) are carbon source such as biodiesel, bioethanol, biofuel etc., and the non-carbon source such as wind energy, solar energy and geothermal. However, it appeared global attention is focused more on carbon sources due to the abundance of biomass. For example, several researchers have extensively studied different types of biomass as potential feed stock for renewable energy under different technologies such as torrefaction, hydrothermal liquefaction, gasification, fast pyrolysis, Co-combustion of renewable biomass and carbonization of solid fuels from lignocellulosic biomass^4–8^. Amongst the products of all these thermochemical conversion technologies, syngas and biooil appears to be of greatest interest. For example, syngas is a basic raw material for chemical industry and transportation fuel with energy density of about 50% of natural gas^5^. On the other hand, biooil which contains several oxygenated groups and fatty acids can be upgraded to wide range of transportation fuels via removal of the oxygen molecules which lowers its overall energy density by about 9–13% compared to conventional diesel fuels^9^. So far, two processing techniques have been studied and reported in the literature for the removal of oxygen molecules from biooil, which are hydrodeoxygenation (HDO, removal of O_2_ as H_2_O) and decarboxylation (removal of O_2_ as CO_2_) or decarbonylation (removal of O_2_ as CO).

In both of these processes, i.e. conversion of biomass to syngas and biooil, certain well designed catalysts are required to produce the final hydrocarbon fuel products that are beneficial to mankind at reasonable process conditions with economical yield. Amongst the well-studied catalyst active metals (and promoters), Ni, Co, Fe, Pd, Pt, Cu, Zr, Ru and Mo have showed varying degree of promising results for both the syngas conversion to higher hydrocarbon fuel in what is referred to as Fischer Tropsch synthesis (FTS), and biooil upgrading to (normal and isomerized paraffinic) hydrocarbon fuels. However, the high cost of Ru, Pd, Pt and sometimes Zr has precluded their application from industrial scale, thus constraining their studies to laboratory or bench scale studies, for example Pd is about 1000 times more expensive than Ni^10^. According to literature survey on FTS^11–14^, Co appears to be preferably studied compared to Fe due to its high selectivity to linear paraffin fractions, slow deactivation, less oxygenates and low water–gas shift (WGS) activity. In these studies, metal-support-interaction (MSI) receives considerable attention since it has strong bearing on the Co dispersion on the surface of the support and the Co-oxide reducibility. Xiong *et al*.^11^ specifically reported that high dispersion of active Co on the surface of support requires some interaction between Co species and the support, and the existence of such interaction is needed to stabilize the catalyst against aggregation of active Co and deactivation under FTS conditions. They concluded that strong MSI decreases both the reducibility and activity of cobalt catalyst under FTS reaction conditions. Similarly, previous studies on the catalytic upgrading of fatty acids and biooil into biofuels either through the HDO or decarboxylation/decarbonylation (Decarbs) routes also showed that Co is essentially a promising active metal. For example, Wu *et al*.^15^ reported decarboxylation of stearic acid over a series of synthesized 20% Ni/Al_2_O_3_, 20% Cu/ZrO_2_, 20% Co/ZrO_2_ and 20% Ni/ZrO_2_. In the report, 20% Co/ZrO_2_ showed about two times stearic acid conversion compared to other catalysts, although its selectivity to the expected product – heptadecane – was low. Another study^16^ compares the conversion of guaiacol and product yields over Al-MCM-41 supported Ni, Co, and Ni–Co catalysts at the same experimental conditions and 10Co/Al-MCM-41 exhibited the highest HDO activity with least tendency towards gas phase yields for CH_4_, CO and CO_2_. In these studies, the challenges to catalyst designs and activities in upgrading fatty acids/biooil to transportation hydrocarbon fuels are similar to what were reported for the FTS which are active metal dispersion, active metal reducibility and proper MSI.

To solve this problem, different studies such as effects of support, promoters, cobalt precursor and synthesis conditions have been explored^11, 13, 14, 17, 18^. In a study on the effect of support, acidic HZSM zeolite appears to be more prospective than alumina due to the propensity of formation of cobalt aluminate which is very difficult to reduce, in addition, HZSM zeolite enhanced the selectivity of the FTS towards gasoline^12^. Some earlier reports^19, 20^ also established that acidic zeolite as a catalyst support or in combination with some other conventional support enhanced selectivity to gasoline range fuel. Co supported on TiO_2_ also showed similar observation to the cobalt aluminate forming cobalt titanates with high Co dispersions but strong Co–support interaction leading to poor Co-oxides reducibility. On the contrary, Co supported on SiO_2_ showed a weaker MSI which favors the reducibility of the cobalt oxides however, the Co agglomerates after calcination process leading to a low dispersion of the Co° particles on the SiO_2_ surface^20^. Consequently, the final surface density of active Co° sites will be a function of degree of cobalt dispersion and reducibility. Report on the effect of promoters such as Ru showed that small amount of Ru promoter to Co/SBA-15 shifted the reduction temperature of Co_3_O_4 → _CoO and CoO → Co to lower temperatures and suppressed the formation of Co^3+^ species^11^. Another study on the influence of cobalt loading (10–40 wt% Co) and effect of Co precursor using Co nitrate, Co acetate and Co acetylacetonate^20^ showed that while using Co nitrate, increase in the Co loading resulted into decrease in the degree of dispersion but increase in the extent of cobalt reduction. Their best observed Co loading for CO conversion was 30%. On the influence of type of precursors, they found that Co catalysts prepared from both acetate and acetylacetonate precursors have higher degree of Co dispersion and a stronger cobalt–support interaction leading to the formation of low reducible cobalt silicates compared to that derived from cobalt (II) nitrate according to their TEM, XPS, and TPR. From all the literature studied, it appeared that high degree of active metal dispersions at the highest possible Co loading and high reducibility of the active species are the most significant factors that affect both CO conversion and selectivity to C_5+_, as well as HDO and Decarbs activity of fatty acids/biooil into hydrocarbon transportation fuel range. However, active metal reducibility has been shown to have more overriding influence than the degree of active metal dispersions since both processes requires metallic Co (i.e. Co^0^) as the active metal state^10, 12, 19–21^. To achieve high active metal reducibility, Al-Dalama and Stanislaus^22^ studied the influence Ethylenediaminetetraacetic acid (EDTA) on the reducibility and metal–support interactions of SiO_2_–Al_2_O_3_ supported mono- and bi-metallic (Ni, Mo and NiMo) catalysts using temperature programmed reduction (TPR) technique, they reported that functionalization of the metals with EDTA reduced the MSI thus enhancing the metal reducibility by lowering reduction temperature from >800 °C to 450–550 °C. Since reduction of metal oxide to metallic state at high temperature is typically prone to sintering of the active metals, it is important to explore other means to synthesize easily or readily reducible supported metal catalysts for FTS and HDO process.

Information regarding the application and suitability of chelating agents to enhance reducibility of Co supported catalysts in FTS and HDO is rarely found in the present literature. Consequently, in this study, cobalt (II) oxalate was synthesize as FTS catalyst precursor via the functionalization of cobalt nitrate with oxalic acid (OxA) as a chelating agent. The prepared Co precursor was subsequently incorporated into ZSM-5 zeolite as catalyst support since ZSM-5 is known to favor gasoline range fuel. The reducibility and other characterization results and activity of the synthesized catalyst were compared with another Co-supported ZSM-5 catalyst without OxA functionalization.

## Materials and Methods

### Materials

All chemicals were purchased from Sigma Aldrich except oxalic acid (OxA) that was purchased from Spectrum chemicals, and all the chemicals were used with any pretreatment.

### Catalyst synthesis

Two different catalyst were synthesized, the first was ZSM-5 zeolite (protonic form) supported cobalt (Co/ZSM-5) and the second was ZSM-5 supported cobalt oxalate (CoOx/ZSM-5) catalyst. The CoOx/ZSM-5 catalyst (30% Co loading) was synthesized in two steps, in the first step, the cobalt (II) oxalate precursor (CoOx) was synthesized via functionalization of 29.59 g cobalt nitrate hexahydrate (dissolved in 50 mL deionized water) with 9.148 g anhydrous OxA (equation (1)) in a round bottom flask wrapped with aluminum foil at 60 °C for 30 min. The observed pH of the CoOx was 1.43. In the second step, all the CoOx was added to 20 g ZSM-5 dispersion in 120 mL of deionized water under continuous stirring at 70 °C for 6 h to ensure complete incorporation of the CoOx into the ZSM-5 support. The Co/ZSM-5 (30% Co loading) was synthesized following the same procedure but without OxA functionalization. The observed pH of the Co precursor was 4.9. Both catalysts were dried in the oven at 100 °C for 12 h, grinded and calcined in nonreactive (N_2_) environment at 450 °C for 4 h.

1 $${\rm{Co}}{({{\rm{NO}}}_{3})}_{2}\cdot 6{{\rm{H}}}_{2}{\rm{O}}+{{\rm{C}}}_{2}{{\rm{H}}}_{2}{{\rm{O}}}_{4}\to {{\rm{CoC}}}_{2}{{\rm{O}}}_{4}+2{{\rm{HNO}}}_{3}+6{{\rm{H}}}_{2}{\rm{O}}$$

### Catalyst characterization

Thermal gravimetric analysis (TGA) was carried out with a SHIMADZU DTG-60/60 H instrument to determine the heat treatment required during calcination. 2 g of each sample was heated in a silica crucible at a constant heating rate of 10 °C/min operating in a stream of N_2_ atmosphere with a flow rate of 40 mL/min from 30 to 800 °C and the weight loss per time, weight loss per temperature increment and temperature increment versus time were recorded. Nitrogen adsorption–desorption measurements (BET method) were performed at liquid nitrogen temperature (−196 °C) with an autosorb BET apparatus, Micromeritics ASAP 2020, surface area and porosity analyzer to determine the surface area, pore size and structure, and the pore volume. Extreme high resolution field emission scanning electron microscope (XHR-FESEM) and energy dispersive X-ray (EDX) were performed to determine the samples morphology and elemental composition, respectively using Verios XHR-FESEM (Model 460 L, FEI™) equipped with a EDX detector. The catalyst microstructure and crystallographic information were studied by high resolution transmission electron microscope (HRTEM) 200 kV with Field Emission (TECNAI G2 20 S-TWIN, FEI™). X-ray diffraction (XRD) patterns of the samples were measured with Philip PW 1820 diffractometer to determine the crystal phase and structure of the incorporated metal. XPS analyses (Thermo-Fischer K-Alpha) were carried out to obtain the chemical nature, surface composition, oxidation state, relative surface compositions and the type of interaction between metal and support. The XPS was equipped with monochromatised AlKα source and the resulting samples spectra were analyzed using the Avantage software for peak fitting and identification of chemical state. The reduction behavior and active metal dispersion of the catalysts was studied using a Thermo Finnigan TPD/R/O 1100 equipped with a thermal conductivity detector and a mass spectrometer. Typically, 20 mg catalyst was placed in the U-shaped quartz tube. Catalyst samples were degassed under a flow of nitrogen at 200 °C to remove traces of water and impurities from the catalyst pores. H_2_ temperature program reduction (TPR) was performed using 5% H_2_/N_2_ with a flow rate of 20 ml min^−1^ and heating from 40 to 700 °C at 5 °C min^−1^. The acidity of the catalysts was analyzed by using temperature programmed desorption (NH_3_-TPD). The sample was pretreated at 300 °C for 2 h in He and cooled to 50 °C. After being saturated with NH_3_, the sample was purged with He to remove the physisorbed NH_3_. The TPD measurements of desorbed NH_3_ were conducted in flowing He from 60 °C to 970 °C at a heating rate of 10 °C min^−1^. All flow rates of He mentioned above were set to 20 mL min^−1^.

### Catalytic performance test

#### Fischer–Tropsch synthesis

The FTS reaction was performed in a stainless steel tube fixed-bed microreactor (PID Eng & Tech). In a given experiment, the stainless steel tube fixed-bed microreactor was disassembled and 0.3 g of either Co/ZSM-5 or CoOx/ZSM-5 catalyst was carefully sandwiched between quartz wools to ensure an isothermal zone around the catalyst and loaded into the stainless steel reactor tube. The catalyst was then reduced *in-situ* in a H_2_ stream at 50 mL min^−1^ at 450 °C for 2 h based on the TPR analysis. During the CO hydrogenation reaction, a mixture of reactant gas comprising H_2_/CO = 2/1 (v/v) was introduced into the reactor. The reaction temperature and pressure were 240 °C and 2 MPa, respectively at a gas hourly space velocity (GHSV) of 1000 h^−1^ for 60 h. All post-reactor lines and valves were heated to 180 °C to prevent condensation. The feed and gaseous products were analyzed by an on-line gas chromatograph (Agilent 7890 A) equipped with two TCD detectors as well as HayesepQ and molsieve columns for analyses of H_2_ and permanent gases. The hydrocarbons and other products were analyzed using a DB-1 column and a FID detector.

#### Oleic acid hydrodeoxygenation experiments

Oleic acid (OA) was hydrodeoxygenated in a 100 mL high pressure semi-batch reactor. The catalysts activities on the HDO process were tested at the previously^2, 23^ best observed reaction conditions of 360 °C and 20 bar for temperature and pressure, respectively. The flow of carrier gas and reaction pressure inlet and outlet were controlled by a flow (Brooks 58505 S) and a pressure controller (Brooks 5866), respectively. Prior to the HDO process, 20 mg of catalyst (Co/ZSM-5 or CoOx/ZSM-5) was reduced under 10 bar H_2_ at 450 °C for 2 h prior to use. In a typical experiment, 40 g (~45 ml) of OA was added to the reactor containing the reduced catalyst after which the reactor was purged with He gas for 10 min. The operating temperature was established and monitored by a type-K Omega thermocouple placed inside the reactor. Before the reaction started, 100 ml/min of 90 vol% N_2_ and 10 vol% H_2_ was passed to the reactor until the desired reaction pressure was reached and the reaction commences by turning on the stirrer at an earlier predetermined speed of 2000 rpm. Based on preliminary studies, all experiments were performed under 60 min and the reactor set up was cooled by forced air before dismantled for product analysis. Liquid samples withdrawn from the reactor were dissolved in pyridine and thereafter silylated with (100 wt% excess of) N,O-bis(trimethyl)-trifluoroacetamide, BSTFA in an oven at 60 °C for 1 h prior to GC analysis. The internal standard eicosane, C_20_H_42_ was added for quantitative calculations. The withdrawn samples were analyzed with a gas chromatograph (GC, HP 6890) equipped with DB-5 column (60 m × 0.32 mm × 0.5 mm) and a flame ionization detector. 1 μl sample was injected into the GC with split ratio of 50:1 and helium was used as the carrier gas. The chromatographic program was well-adjusted to achieve satisfactory separation of the desired product and the product identification was validated with a gas chromatograph–mass spectrometer (GC–MS). OA molar conversion was calculated by dividing reacted moles by the initial number of moles loaded into the reactor, while the selectivity (S) was calculated as the number of moles of product recovered divided by the number of moles of OA that had reacted. To minimize uncertainties and guarantee reproducibility, some of the data point were repeated in three independent experiments. All data generated or analyzed during this study are included in this published article.

## Results and Discussions

### Catalyst characterization

#### Thermal gravimetric analysis

**Figure 1:**
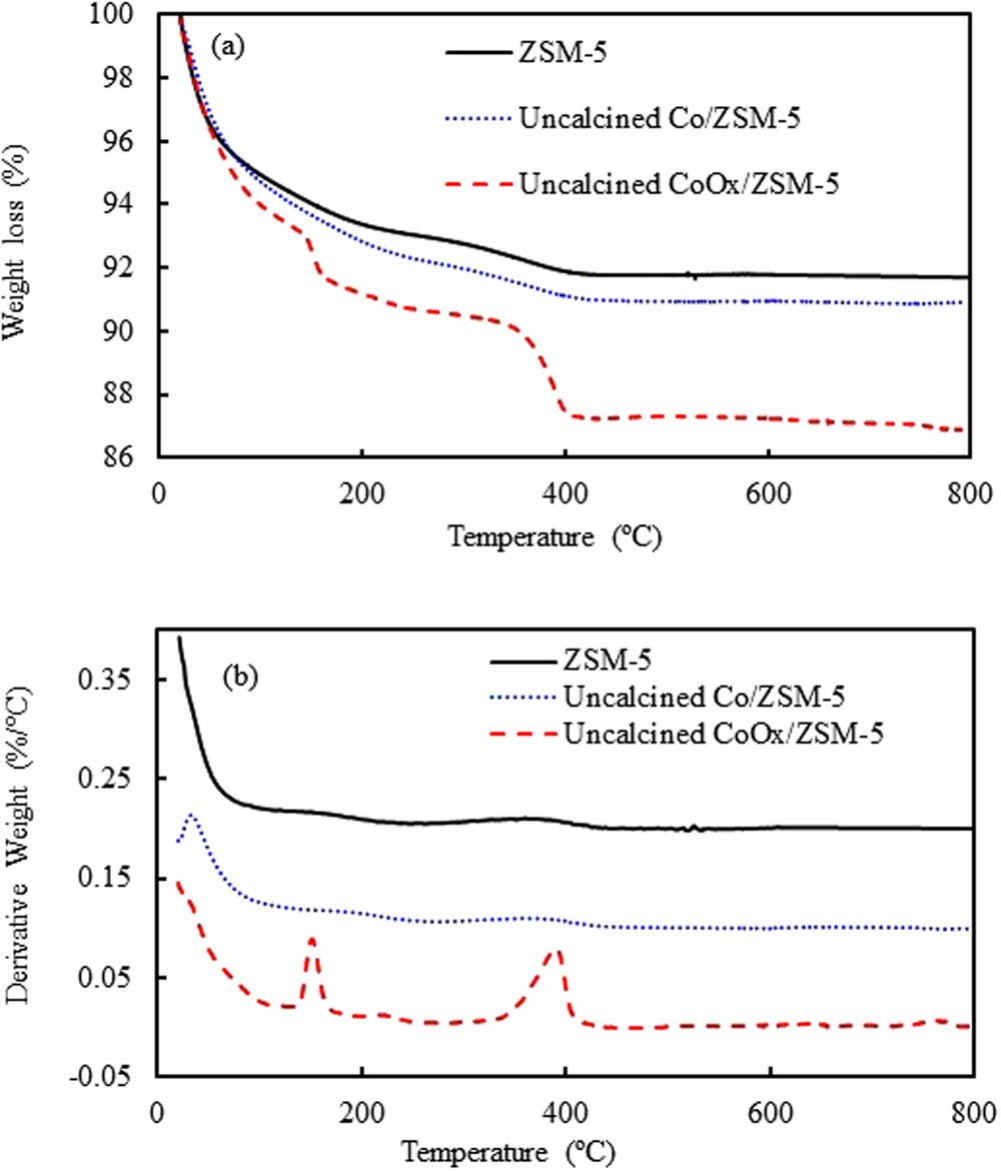
TGA-DTG curves obtained for ZSM-5, Co/ZSM-5 and CoOx/ZSM-5 catalysts.

Generally, calcination temperature has a critical influence on the texture and crystallite size of supported catalyst based to the thermal response of the active metal precursors such as Co_3_O_4_ and hydrated CoOx (CoC_2_O_4_·2H_2_O) which in turn will affect the activity of the synthesized catalysts. Consequently, it is imperative to study the thermal behavior of the synthesized catalysts in relation to each other, and of course to the support. The TGA and DTA profiles of ZSM-5, Co/ZSM-5 and CoOx/ZSM-5 samples are shown in Fig. 1a,b. According to previous reports^24, 25^ aluminosilicates generally shows three different and significant weight loss regions (WLR) under thermal treatment. From Fig. 1a, the first weight loss region below 180 °C was ascribed to interlayer and physisorbed water molecules which are mobile and freely bounded^24, 25^. The second WLR usually in the range of 300–500 °C are attributed to strongly bonded water molecules that are present in the first coordination sphere of the interlayer ions or lattice structure. The third weight loss at temperatures above 650 °C is characteristics of the structural hydroxyl groups that will condense and dehydrate at elevated temperatures^26^. Up to about 70 °C, all the three samples showed almost equal weight loss of 4.0%, however as the temperature ramped up to about 140 °C, Co/ZSM-5 showed further increase in weight loss with a cumulative of ~7.0% which can be ascribed to the interlayer and physisorbed water molecules as well as hydration effect during catalyst synthesis stage. Meanwhile, CoOx/ZSM-5 showed two regions with increased weight loss within the first WLR and maximized at 154 °C according to Fig. 1b with a cumulative weight loss of 9.2% which can be ascribed to combined effect of the interlayer/physisorbed water molecules, hydration effect at the catalyst synthesis stage and complete dehydration of incorporated CoOx precursor (CoC_2_O_4_·2H_2_O) as shown in equation (2). From the DTA profile in Fig. 1b, there is a low intensity broad peak with maxima at 366 °C corresponding to 1.34% and 1.4% weight losses (Fig. 1a) for ZSM-5 and Co/ZSM-5, respectively in the second WLR which was earlier ascribed to strongly bonded water molecules. However, CoOx/ZSM-5 showed a distinctive peak at 383 °C and this was ascribed to combined loss of strongly bonded water molecules as seen in the other samples (ZSM-5 and Co/ZSM-5) as well as thermal decomposition of the already dehydrated CoC_2_O_4_ into metallic Co and CO_2_ as shown in equation (3)^27, 28^. The observed weight loss in this region was ~3.5% which implied that about 2.1% was due to thermal decomposition of CoC_2_O_4_ in the ZSM-5 matrix. This observation is in agreement with a recent study on the effect of microwave power on the thermal genesis of unsupported Co_3_O_4_ nanoparticles from cobalt oxalate micro-rods where a considerable weight loss with a sharp exothermic effects at 354 °C was reported^29^.

2 $${{\rm{CoC}}}_{2}{{\rm{O}}}_{4}\cdot 2{{\rm{H}}}_{2}{\rm{O}}\to {{\rm{CoC}}}_{2}{{\rm{O}}}_{4}+2{{\rm{H}}}_{2}{\rm{O}}$$

3 $${{\rm{CoC}}}_{2}{{\rm{O}}}_{4}\to {\rm{Co}}+2{{\rm{CO}}}_{2}\,\,\,$$

#### Nitrogen adsorption/desorption isotherms

**Figure 2:**
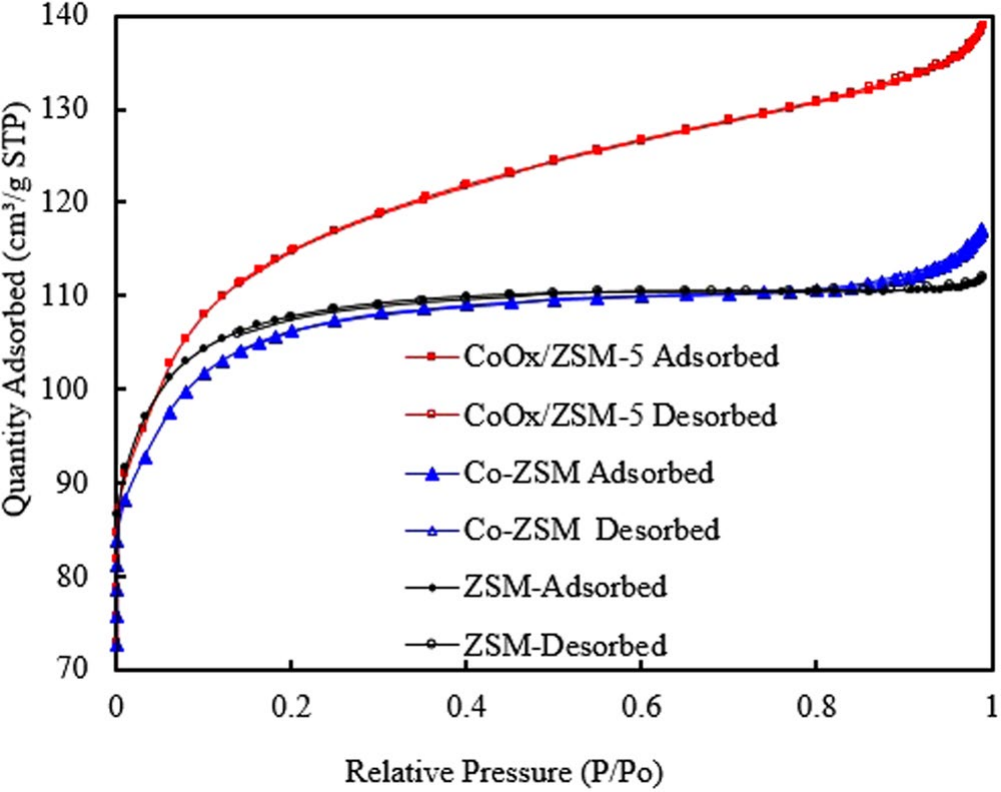
N_2_ adsorption–desorption isotherms of ZSM-5, Co/ZSM-5 and CoOx/ZSM-5.

The N_2_ adsorption-desorption isotherms in Fig. 2 clearly shows that the volume of N_2_ adsorbed increases obviously with increasing relative pressures (P/P_o_) for ZSM-5, Co/ZSM-5 and CoOx/ZSM-5, and this can be attributed to the volume filling of micro-pores in the samples^30^. According to the isotherm of the samples, the volume of N_2_ adsorbed followed the order CoOx/ZSM-5 > ZSM-5 > Co/ZSM-5. The textural properties of all the three samples are summarized in Table 1. The external surface area and volume as well as the micropore area and micropore volume were calculated by the t-Plot method, and surface area was calculated by the (Brunauer-Emmett-Teller) BET method. The enhancement in the surface area, pore volume and pore size of CoOx/ZSM-5 catalyst was due to the leaching influence of the acidic CoOx precursor and protonic effect from HNO_3_ (equation (1)). Previous reports^12, 30, 31^ have shown that acid modification/treatment of aluminosilicates enhances textural properties such as porosity, specific surface area and pore volume via acid leaching of framework and extra-framework aluminum species. The well-developed external surface area and enhanced porosity are considered advantage towards guaranteeing high active metals dispersion thus inhibiting the propensity of sintering and formation of bulk Co-oxide. Conversely, the reduction in the specific surface area, pore volume and pore size in the Co/ZSM-5 was attributed to the partial blocking of the pores with bulk oxidized Co-oxide from the thermal decomposition of Co(NO_3_)_2_ precursor according to equation (4). The oxidized Co_3_O_4_ with increased particle sized having strong interaction with the ZSM-5 support was also verified by the HRTEM, XHR-SEM and XPS results.

4 $$3({\rm{Co}}{({{\rm{NO}}}_{3})}_{2}\cdot 6{{\rm{H}}}_{2}{\rm{O}})\to {{\rm{Co}}}_{3}{{\rm{O}}}_{4}+6{{\rm{NO}}}_{2}+{{\rm{O}}}_{2}+18{{\rm{H}}}_{2}{\rm{O}}$$

**Table 1:** Textural properties of ZSM-5, Co/ZSM-5 and CoOx/ZSM-5.

Sample	t-Plot External Surface Area (m^2^/g)	t-Plot Micropore Area (m^2^/g)	BET Surface Area (m^2^/g)	total pore volume (cm^3^/g)	t-Plot External volume (cm^3^/g)	t-Plot micropore volume (cm^3^/g)	Pore diameter (nm)
ZSM-5	98.5	282.49	381.0	0.178	0.058	0.12	1.98
Co/ZSM-5	73.2	274.6	347.9	0.17	0.04	0.11	1.88
CoOx/ZSM-5	151	258.02	409.1	0.21	0.10	0.13	2.07

#### EDX, TEM and XHR-SEM

**Figure 3:**
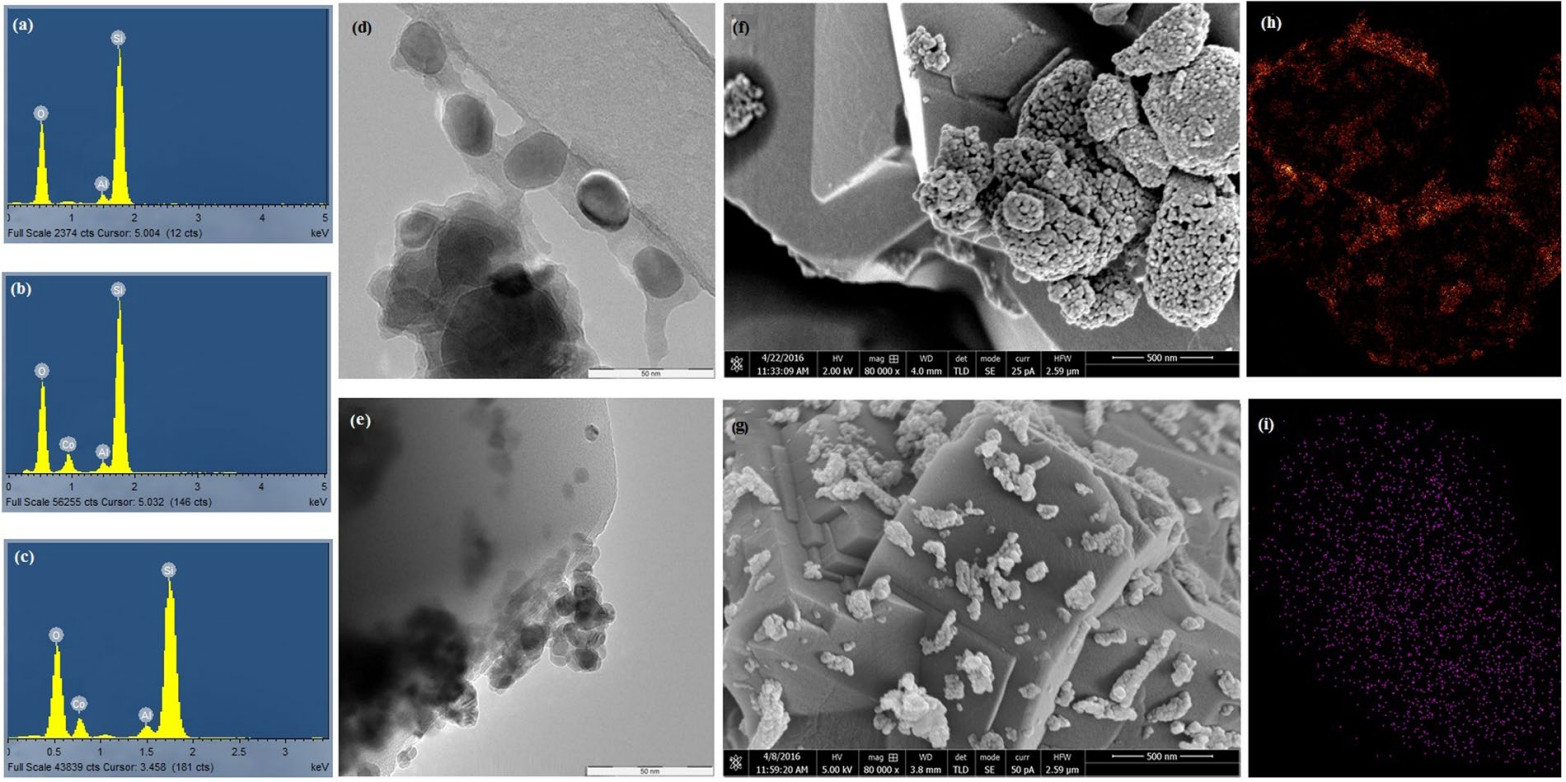
(**a**–**c**) EDX spectra of ZSM-5, Co/ZSM-5 and CoOx/ZSM-5, (**d**,**e**) TEM image of Co/ZSM-5 and CoOx/ZSM-5, (**f**-**g**) XHR-FESEM images of Co/ZSM-5 and CoOx/ZSM-5, (**h**–**i**) EDX mapping of Co/ZSM-5 and CoOx/ZSM-5.

The EDX spectrum in Fig. 3a shows a high Si/Al ratio of 20 according to the quantification of the elemental analysis with average crystallite size is 1.85 micrometer (see Supplementary Figure S1). The spectra of Co/ZSM-5 and CoOx/ZSM-5 in Fig. 3b-,c confirmed the successful incorporation of the respective Co precursors. Figure 3d,e show the HR-TEM images of Co/ZSM-5 and CoOx/ZSM-5 catalysts, respectively. The results revealed instances of agglomeration of metal particles in Co/ZSM-5 catalyst possibly due to sintering of the active metals during calcination. Conversely, CoOx/ZSM-5 shows a clear degree of active metal dispersion. In addition the average metal particles sizes are smaller in CoOx/ZSM-5 than in Co/ZSM-5 which can also be ascribed to the acidic effect of OxA functionalization at the synthesis stage. Fakeeha *et al*.^12^ reported that both active metal dispersion and metal particle size are very important factors in catalysts activity and controlling of carbon deposition. From their studies, catalysts with large particle sizes exhibits comparably lower activity and suffer acute carbon deposition, while catalysts with smaller particles sizes showed higher activities. Other studies have also shown that the modification of active metals with organic substance such as EDTA^22, 32^ and OxA^1, 26^ guarantee active metal dispersion with comparably smaller particles sizes leading to increased activity. Of special importance is the report of Martínez *et al*.^20^ on the influence of cobalt precursor for the synthesis of Co/SBA-15 catalyst, their TEM result supported by the XPS and TPR results showed that a much better Co dispersion was observed for oxidized samples prepared from acetate and acetylacetonate organic precursors as compared to that derived from cobalt (II) nitrate precursor. As previously noted, the enhancement in the textural properties of CoOx/ZSM-5 (Table 1) was ascribed to the acidic CoOx precursor and protonic effect from HNO_3_ which increased its surface area for effective Co dispersion, while the reduction in the pore volume and surface area of Co/ZSM-5 was ascribed to the blockage of the pores by the agglomerated Co particles as seen in Fig. 3d. The XHR-SEM morphology of Co/ZSM-5 in Fig. 3f further supported the agglomeration of Co while reasonable active metal dispersion is very obvious from the morphology of CoOx/ZSM-5 in Fig. 3g. The variation in the Co particles sizes in both catalysts is attributed to differences in metal support interaction due to the Co ligands from the functionalization of OxA which acts as structure directing agent (SDA) for incoming Co to interact with the support rather than with already formed Co cluster in CoOx/ZSM-5. The EDX mapping of Co/ZSM-5 and CoOx/ZSM-5 in Fig. 3g,h also confirmed that Co particles suffers agglomeration in the former while there is ample Co dispersion in the latter.

#### X-ray photoelectron spectroscopy

**Figure 4:**
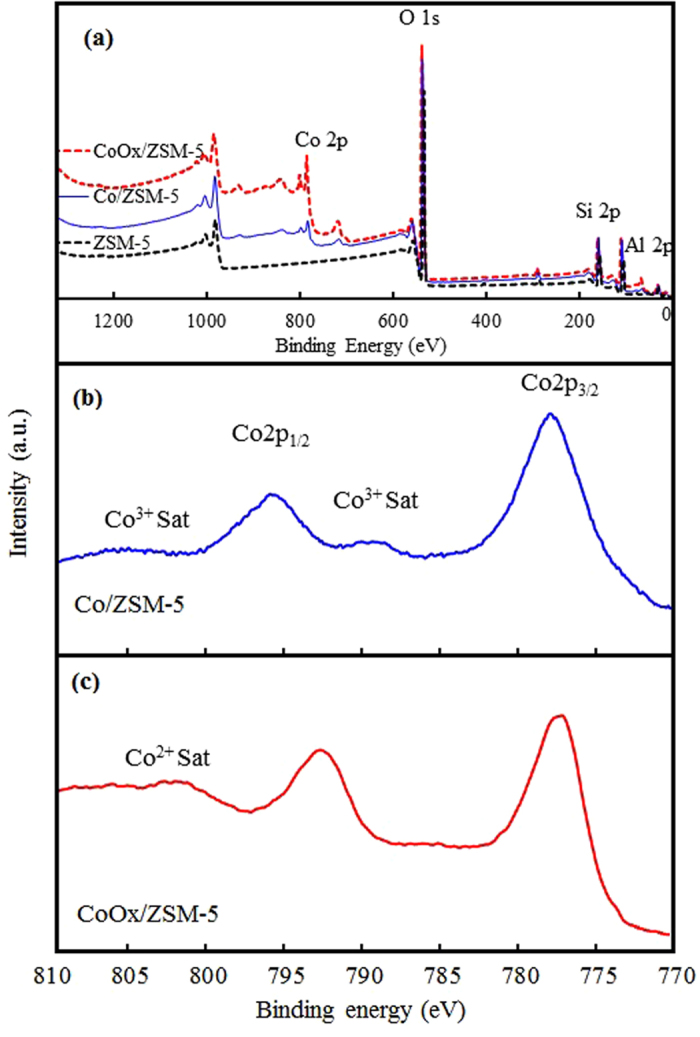
(**a**) Wide-survey XPS spectra scan of ZSM-5, Co/ZSM-5 and CoOx/ZSM-5, (**b**) Co 2p spectra of Co/ZSM-5, (**c**) Co 2p spectra of CoOx/ZSM-5.

The results of XPS analysis performed to determine the variations in the oxidation states of Co based on the effect of OxA functionalization are shown in Fig. 4 for Co/ZSM-5 and CoOx/ZSM-5 catalysts. The survey scan of the ZSM-5 support and the catalyst are shown in Fig. 4a, the ZSM-5 survey scan showed the presence of Al, Si and O at binding energy (BE) of 67.35 eV, 107.38 eV and 537.89 eV, respectively. Both Co/ZSM-5 and CoOx/ZSM-5 revealed the presence of Co particles at BE of 796.45–778.68 eV. These peaks are consistent with the EDX results, and in agreement with previous literature reports^30, 32^. From Fig. 4b, the Co/ZSM-5 catalyst showed BE at 779.88 eV and 796.18 eV, respectively representing the Co 2p_3/2_ and Co 2p_1/2_ with a satellite peak at 791 eV and BE split ∆E_Co_ (i.e. ∆E_Co = _Co 2p_1/2_ − Co 2p_3/2_) of 16.2 eV which is typical of Co^3+^ from the mixed-valance Co_3_O_4_. Therefore, Co_3_O_4_ was considered the cobalt phase in Co/ZSM-5 catalyst^12^. However, for the CoOx/ZSM-5 catalysts (Fig. 4c), the Co 2p_3/2_ and Co 2p_1/2_ shifted towards lower BE of 778.21 eV and 793.55 eV, respectively, with comparably lower ∆E_Co_ of 15.35 eV. The BE of 778.21 eV at Co 2p_3/2_ peak is typical of Co metal which showed that Co metal is present in the CoOx/ZSM-5 catalysts. This is further supported by the absence of distinct satellites shake-up peaks between Co 2p_3/2_ and Co 2p_1/2_ ascribable to either Co2^+^ or Co^3+^ oxidation states, however, a satellite at 802.3 eV suggests the presence of Co^2+^ species^12, 30, 32^. The presence of Co metallic state has been ascribed to the thermal decomposition of cobalt oxalate under inert atmosphere as shown in equation (3)^27^ while the inadvertent present of Co^2+^ can be ascribed to re-oxidation of the metallic Co according to equation (5) from the CO_2_ produced in equation (3)^28^. This confirmed that the chemical state of Co is greatly influenced by the OxA functionalization in the CoOx/ZSM-5 catalyst. Therefore, it can be summarized that the Co/ZSM-5 catalyst contains mainly Co^3+^ from Co_3_O_4_ as dominant species whereas the CoOx/ZSM-5 catalyst contains Co^0^, and Co^2+^ ions in ion-exchange positions i.e. extra-framework Co^2+^ ^32^.

5 $${\rm{Co}}+{{\rm{CO}}}_{2}\to {\rm{CoO}}+{\rm{CO}}$$

This was further corroborated by the shift in the Co 2p_3/2_ and Co 2p_1/2_ peaks at BE of 778.2 eV and 793.55 eV in CoOx/ZSM-5 to higher BE of 779.88 eV and 796.18 eV, respectively in Co/ZSM-5 which implied that there is very close assembly between Co_3_O_4_ particles and the ZSM-5 support leading to stronger metal support interaction (MSI) in Co/ZSM-5 catalyst. Thus, the OxA functionalization has been effective in guaranteeing surface active Co particles by reducing Co^2+^ and further constrained its electro-oxidation during the catalyst synthesis stage. It is well known that oxalate anion (C_2_O_4_^2−^) is a versatile chelating ligand that forms solid dihydrate chains with several divalent metals such as Zn, Mg, Mn, Fe, Ni, and Co. In these metal complexes, the oxalate molecule adopts a planar (D_2h_) configuration such that the O–O distance adjusts to best match the cationic size which makes oxalate a very versatile adsorbate and reducing compound. Thus, the oxalate anion is planar or near planer when bonded to small cations, and when bonded to larger cations the oxalate anion becomes twisted^26^. In this case, as CoOx solution complexes, the oxalate anion adjusts its dihedral angle, and therefore the O–O spacing to match the size and spacing of the Co substrate leading to its adsorption on Co which minimized the access of Co to oxidative environment. This explains why there was no signature of Co^3+^ species at all in CoOx/ZSM-5 catalyst. In addition, the enhanced surface area and porosity earlier seen in the BET result also ensured adequate active metal dispersion thus preventing agglomeration of active metal or sintering during calcination stages which can lead to formation of bulk Co-oxide with strong MSI as seen in the Fig. 3f. On the contrary, the reduction in the surface area of Co/ZSM-5 catalyst was ascribed to the formation of highly oxidized bulk Co-oxide (Fig. 3g) which partially blocked the pores of the support. These observations support the TEM results (Fig. 3d,e) and it explains why the O 1 s/Co 2p ratio in Co/ZSM-5 is higher than in CoOx/ZSM-5 (Table 2).

**Table 2:** XPS data of ZSM-5, Co/ZSM-5 and CoOx/ZSM-5.

Samples	XPS BE (eV)		Atomic ratios	
	Co 2p_3/2_	Co 2p_1/2_	O 1 s/Co 2p	O_lattice/_O_adsorbed_
ZSM-5	—	—	—	0.694
Co-ZSM-5	779.88	796.18	5.67	1.961
CoOx/ZSM-5	778.21	793.55	2.99	0.264

**Figure 5:**
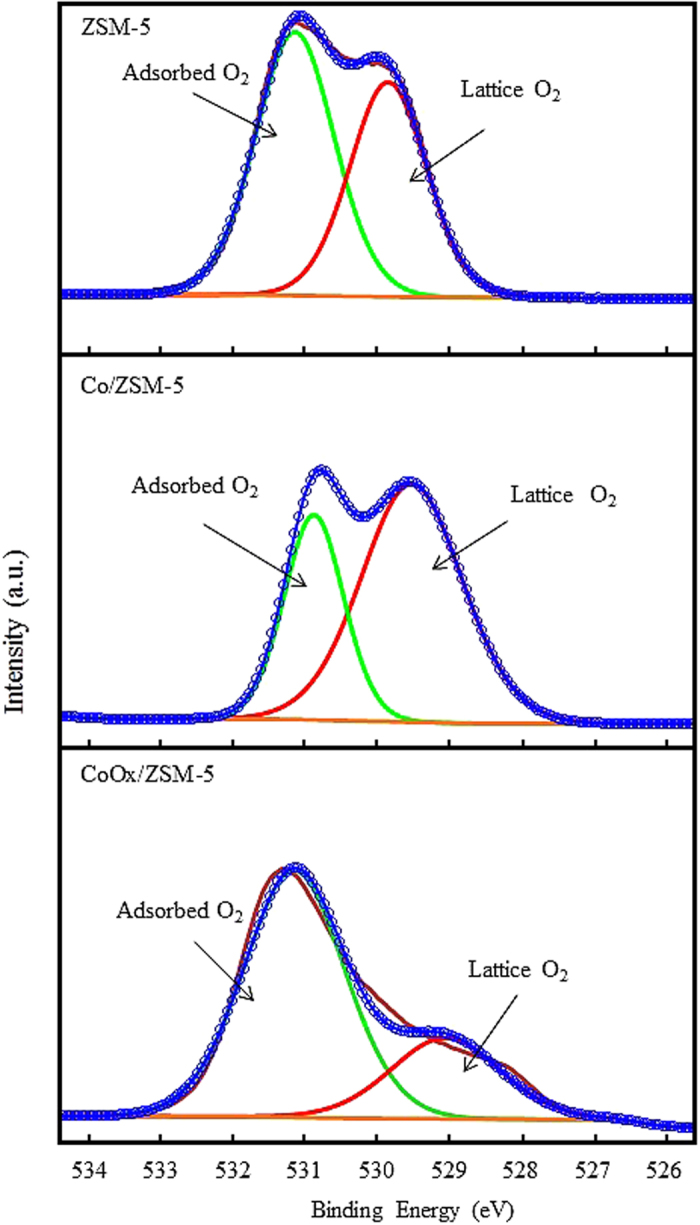
XPS O 1 s spectra of ZSM-5, Co/ZSM-5 and CoOx/ZSM-5.

**Figure 6:**
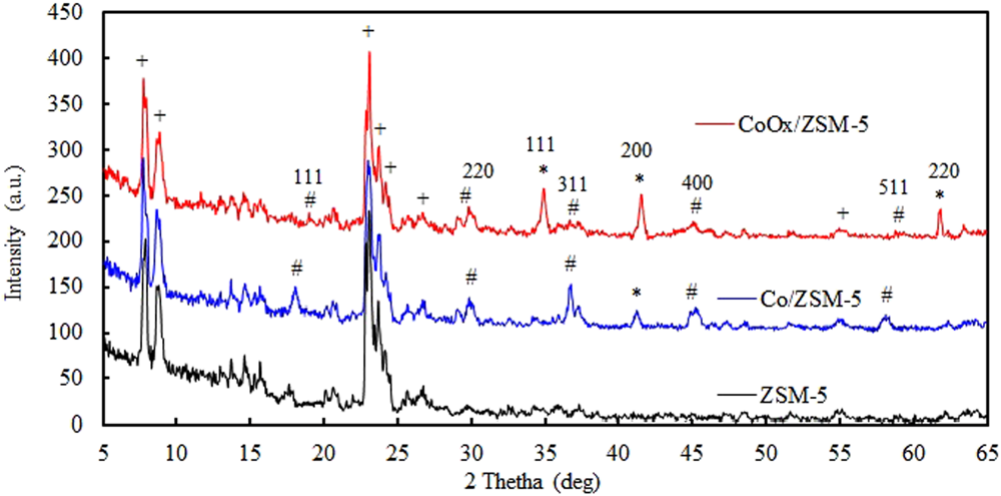
XRD patterns of ZSM-5, Co/ZSM-5, CoOx/ZSM-5.

The XPS spectra of O 1 s for the ZSM-5 support, Co/ZSM-5 and CoOx/ZSM-5 are presented in Fig. 5, all the samples exhibited asymmetric two band structures. The peaks at lower BE of 529 eV are attributed to the lattice oxygen which are bonded to Co particles, while the peaks at higher BE 531.5 eV are characteristic of the adsorbed/surface oxygen, for example, from moisture. Table 2 shows that the ratio of O_lattice_/O_adsorbed_ increased in the order: Co/ZSM-5 > ZSM-5 > CoOx/ZSM-5. The result implied that during the catalysts synthesis stage, the Co particles in Co/ZSM-5 were highly oxidized while the oxalate anion (C_2_O_4_^2−^) ligand in CoOx/ZSM-5 mitigated the oxidation of the Co particles. Obviously, this explains why bulk Co-oxides with larger particle sizes was observed in the Co/ZSM-5 having close assembly and stronger MSI according to the HR-TEM, XHR-SEM and XPS results. This observation is in agreement with the report of Chen *et al*.^30^ on modification of ZSM-5 membranes with Co–Cu–Mn mixed oxides. Since the O_lattice_/O_adsorbed_ ratio is lower in CoOx/ZSM-5 compared to Co/ZSM-5 thus leading to higher O 1 s/Co 2p ratio in Co/ZSM-5 than CoOx/ZSM-5 (Table 2), then, the propensity for effective reduction process with H_2_ gas is more guaranteed in CoOx/ZSM-5. This observation further supports the high resolution Co 2p scan results (Fig. 4b,c) which showed that Co^3+^ from Co_3_O_4_ and combine Co^0^ + Co^2+^ are the predominant species in Co/ZSM-5 and CoOx/ZSM-5, respectively, and it is in agreement with the X-ray diffraction patterns in Fig. 6.

#### X-ray diffraction patterns

XRD patterns of ZSM-5, Co/ZSM-5 and CoOx/ZSM-5 are shown in Fig. 6 with distinctive ZSM-5 peaks at 2θ = 7.8°, 8.9°, 22.2°, 23.8°, 24.9°, 26.8°, 30.1° and 45.5° ^1, 30^. It is clearly evident that the intrinsic lattice structure of the ZSM-5 support in both catalysts was sparingly conserved. Although their diffraction peaks exhibits marginal lower intensities with apparent loss of crystallinity (similar to the observation in their morphologies) after incorporation of respective Co precursors and calcination, especially at lower diffraction angle below 2θ = 20°. The sparing conservation of the intrinsic lattice structure of the ZSM-5 support is an important factor considering the fact that ZSM-5 zeolite has been reported to enhance the selectivity of FTS towards gasoline^12^. This shows that the Co/ZSM-5 and CoOx/ZSM-5 synthesis procedure is sufficiently expedient. The diffraction peaks at 2θ = 18.4°, 31.7°, 36.9°, 44.9° and 59.4° in Co/ZSM-5 catalysts indicated that the cobalt species was mainly in the form of crystalline Co_3_O_4_^11^. These peaks were not present in the CoOx/ZSM-5 samples which support the XPS result that Co^3+^ is the predominant specie in Co/ZSM-5. The peak at 2θ = 42.5° in Co/ZSM-5 catalyst was ascribed to stable CoO species possibly in the pores of the ZSM-5 support or from Co_3_O_4_. The peak is also seen in CoOx/ZSM-5, in addition, there are diffraction peaks at 2θ = 41.3°, 44.4° and 48.7° in CoOx/ZSM-5 which are reflections of the Co^0^ ^33^. This observation confirmed that the oxalate ligand functionalization was able to reduce and constrain the electro-oxidation of cobalt oxidation state due to the versatility of oxalate anion (C_2_O_4_^2−^) chelating ligand that forms stable polymorphic structure with a planar (D_2h_) configuration to constrain the electro-oxidation^26^.

#### Temperature program reduction

**Figure 7:**
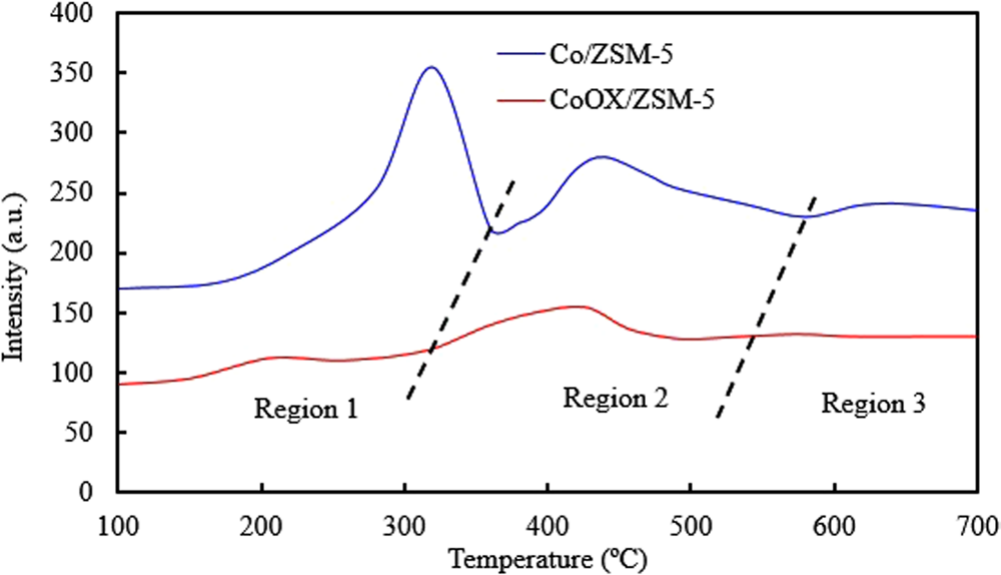
H_2_-TPR profiles of the Co/ZSM-5 and CoOx/ZSM-5 catalysts.

The activity of FTS catalysts have been reported to be strongly dependent on the reducibility of the CoO and Co_3_O_4_ species since metallic Co is responsible for the CO hydrogenation and HDO processes. Consequently there is need for H_2_-TPR studies to validate the effect(s) of OxA functionalization earlier seen in other characterization results as shown in the H_2_-TPR profiles of Co/ZSM-5 and CoOx/ZSM-5 in Fig. 7. The TPR profiles have been normalized per weight of cobalt in the catalyst to facilitate easy discussion. Typically the reduction process of Co_3_O_4_ particles proceeds in two distinctive steps, firstly reduction of Co^3+^ to Co^2+^ at a low temperature and the subsequent reduction of Co^2+^ to Co° at a high temperature as depicted by equations 5 and 6, respectively. From Fig. 7, there are three distinctive regions for Co/ZSM-5 sample, the reduction process in equations 5 and 6 were observed at reduction peak 320 and 440 °C in regions 1 and 2, respectively. There is another high-temperature peak at 650 °C in region 3 which could be assigned to more stable species such as cobalt silicate or cobalt aluminate^20^, or even bulk Co_3_O_4_ (as seen in the TEM result) strongly interacting with the ZSM-5 support as discussed in the XPS results. The reduction peak values observed for Co/ZSM-5 were in close agreement with the report of Wu *et al*.^12^ that shows two distinct reduction peaks around 320 °C and at about 450 °C in the region 1 and 2, and a weak reduction peak at 650 °C in region 3 which was also ascribed to stable species such as cobalt silicate or cobalt aluminate on the ZSM-5 support. The TPR profile of CoOx/ZSM-5 in region 1 showed an inconspicuous hump with a maxima at 205 °C which can be ascribed to reduction of adventitious oxidized Co^3+^ → Co^2+^ (although the presence of these adventitious oxidized Co^3+^ species were not observed in other techniques, it was ascribed to inadvertent exposure of the catalyst sample to atmosphere during sample preparation prior to TPR analysis). Furthermore, the reduction took place at comparable lower temperature which confirmed that they were instantaneous superfluous surface species. The second reduction peak in the region 2 for CoOx/ZSM-5 with a maxima at 420 °C correspond to reduction of CoO (which was earlier ascribed to reduction of CO_2_ according to equation (3) to Co° (equation (6)). The peak shifted towards lower reduction temperature compared to that of Co/ZSM-5 which further confirmed that the OxA functionalization was effective in minimizing the MSI leading to easily reducible species. Both peaks in region 1 and 2 have comparably lower intensities than those of Co/ZSM-5 which implied that the amount of hydrogen gas consumption based on equations (5–6) is low in CoOx/ZSM-5. This supports the observation in the Co 2p spectra of Co/ZSM-5 and CoOx/ZSM-5 in the XPS result and more importantly, it corroborates the comparably higher O 1 s/Co 2p ratio in Co/ZSM-5 than in CoOx/ZSM-5 shown in Table 2.

5 $${{\rm{Co}}}_{3}{{\rm{O}}}_{4}+{{\rm{H}}}_{2}\to 3{\rm{CoO}}+{{\rm{H}}}_{2}{\rm{O}}$$

6 $${\rm{CoO}}+{{\rm{H}}}_{2}\to {\rm{Co}}^\circ +{{\rm{H}}}_{2}{\rm{O}}$$

#### Temperature program desorption-NH_3_

**Figure 8:**
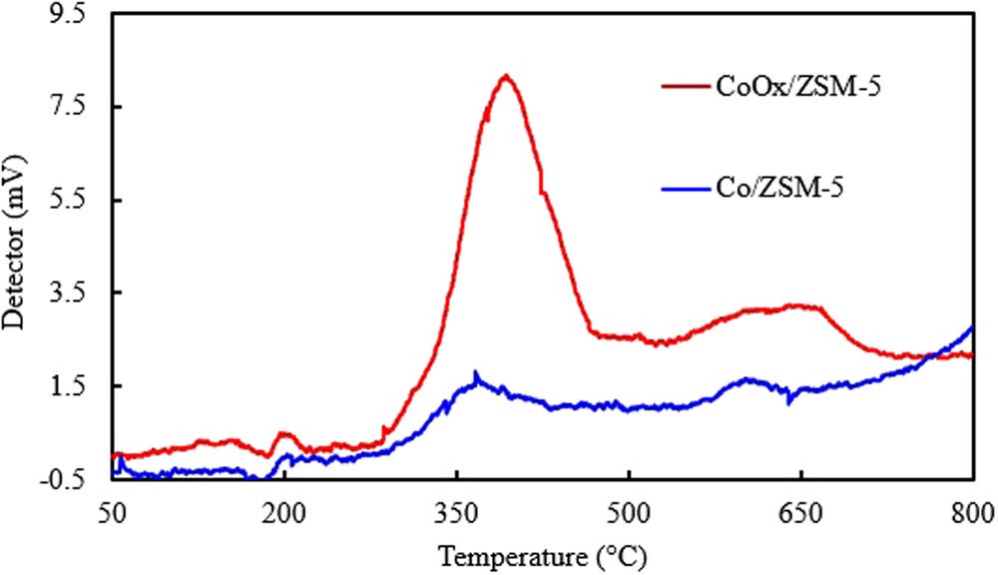
NH_3_ – TPD profiles of the Co/ZSM-5 and CoOx/ZSM-5 catalysts.

The result of catalyst acidity test for Co/ZSM-5 and CoOx/ZSM-5 are shown in the NH_3_- TPD profile in Fig. 8. Co/ZSM-5 revealed two distinctive acid site peaks, one at lower temperature of 376 °C which was assigned to the aluminum centers of ZSM-5 and the dispersion of Co_3_O_4_ on the support of ZSM-5^12^. The peak shifted to higher temperature of 395 °C with increased intensity in CoOx.ZSM-5 and this variation was attributed to higher degree of Co species (Co and CoO) dispersion as well as comparably lower particle sizes (as seen in Fig. 3) leading to higher adsorption and desorption of the physisorbed NH_3_ from the acid sites on the catalyst surface. The second peak in Co/ZSM-5 is at 596 °C which can be attributed to strong acid sites especially in the catalyst pores. The peak became relatively more intense, broader and simultaneously shifted to higher temperature of 620 °C in CoOx/ZSM-5 which implied that there is higher desorption of the physisorbed NH_3_ from the acid sites in the CoOx/ZSM-5 pore but at a comparatively slower rate. These disparities in the Co/ZSM-5 and CoOx/ZSM-5 response to acidity test was ascribed to both the direct effect of acidic influence of oxalic acid functionalization and indirect effects as earlier seen in the BET, HR-TEM, XHR-SEM, XPS and XRD results of CoOx/ZSM-5. According to previous report^12^, the acid sites are promotes isomerization and cracking of heavier hydrocarbons generated on the surface cobalt sites, thus CoOx/ZSM-5 is expected to favor skeletal isomerization of n-paraffin.

### Performance of Co/ZSM-5 and CoOx/ZSM-5 catalysts

#### Fischer–Tropsch synthesis

**Figure 9:**
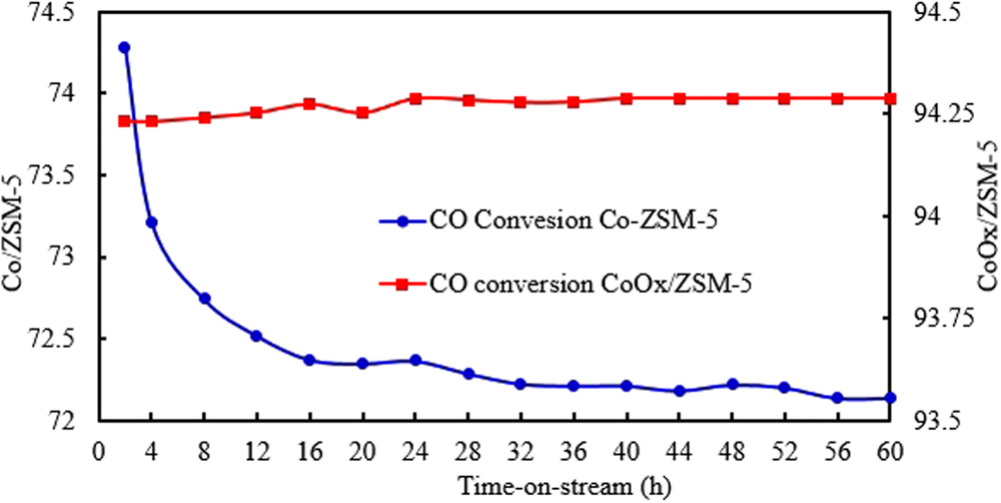
Time-on-stream evolution of CO conversion over Co/ZSM-5 and CoOx/ZSM-5. catalysts. Reaction conditions: T = 240 °C, P = 2.0 MPa, H_2_/CO = 2 and GHSV = 1000 h^−1^.

Time-on-stream (TOS) evolution of CO conversion over Co/ZSM-5 and CoOx/ZSM-5 catalysts shown in Fig. 9 were 74.28% and 94.23% at 2 h TOS. The 74.28% observed for Co/ZSM-5 is in the neighborhood of 70.5% reported by other authors using Co supported on ZSM-5 at similar experimental conditions^12^. Although with increasing TOS, Co/ZSM-5 loss about 2% CO conversion activity within the first 16 h TOS and its activity remains almost unchanged afterwards for the next 44 h TOS. However, the CO conversion activity of CoOx/ZSM-5 was almost unchanged over the entire 60 h TOS. The initial loss of activity in Co/ZSM-5 is similar to the observation of Sartipi *et al*.^21^ using Co/H-ZSM-5 at similar operating condition which showed about 11% loss in CO conversion activity within 6 h TOS due to partial deactivation. The superiority display of CoOx/ZSM-5 was ascribed to the OxA functionalization which enhances its textural properties, degree of dispersion and metal support interaction leading to readily and effective reducibility as seen in the characterization results. In addition, the comparable smaller particle size of Co species leading to more active sites as seen in the TEM result (Fig. 3) for CoOx/ZSM-5 also contributed to its higher CO conversion activity. This is supported by previous reports^12, 22, 26, 32^ which showed that smaller particle sizes generally exhibits higher active metal dispersion and reducibility leading to comparably higher activity due to increase in the number of active sites, while catalysts with larger particles sizes showed poor active metal dispersion and reducibility leading to comparably lower activities.

**Figure 10:**
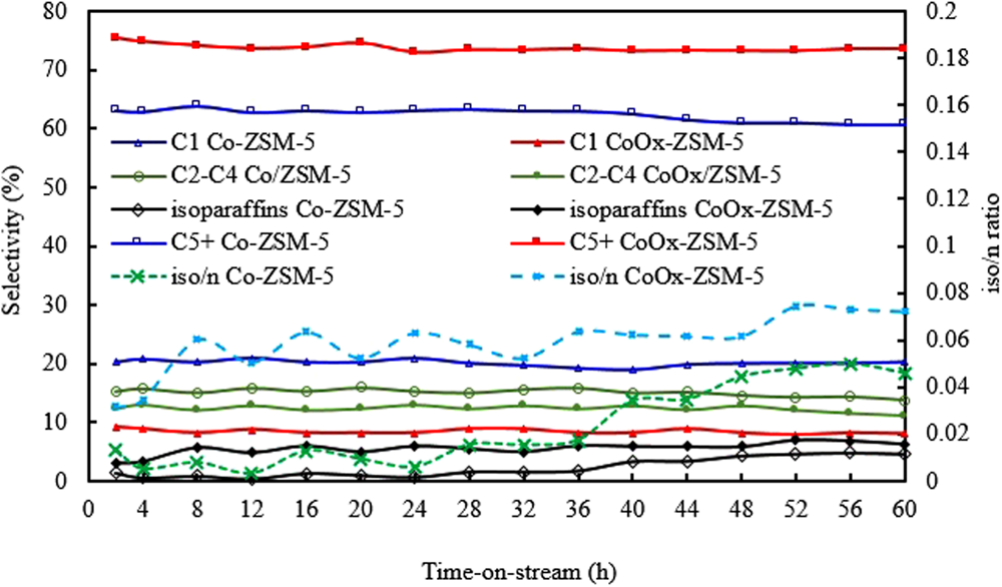
Time-on-stream products selectivity over Co/ZSM-5 and CoOx/ZSM-5 catalysts, Reaction conditions: T = 240 °C, P = 2.0 MPa, H2/CO = 2 and GHSV = 1000 h^−1^.

The selectivity of the products obtained from both catalysts is shown in Fig. 10, with Co/ZSM-5 showing higher selectivity to light hydrocarbon (HC) products (C_1_ – C_4_) and lower selectivity for C_5+_ products. On the other hand, CoOx/ZSM-5 showed inverse relationship with higher selectivity to C_5+_ HC products and lower selectivity for C_1_ – C_4_. The higher selectivity for lower HC especially CH_4_ was due to the comparably poor reducibility of Co_3_O_4_ particles in Co/ZSM-5 since previous study has shown that CoO surface is essentially effective for CH_4_ production^12^. Khodakov *et al*.^13^ also reported an inverse relationship between methane selectivity and the degree of Co reduction in a series of cobalt–supported mesoporous silicas with different pore sizes. Consequently, the higher selectivity to C_5+_ products in CoOx/ZSM-5 was due to the influence of the OxA functionalization that enhanced the textural properties and guarantee the formation of comparably smaller particle size Co^0^ and highly reducible CoO with higher dispersion. This is in agreement with a related study^12^ on the preliminary evaluation of ZSM-5/SBA-15 composite supported Co catalysts for FTS which reported that higher cobalt dispersion and increase in supported catalyst porosity are essential to maximize the formation of the C_5+_ hydrocarbons. The study showed that as the amount of ZSM-5 is increased in the ZSM-5/SBA-15 composite from 0% to 30%, the C_5+_ hydrocarbons increase from 65.1% to 79.3% and they ascribed this increment to higher cobalt dispersion and enhanced catalyst pore sizes.

It is worth noting that both Co/ZSM-5 and CoOx/ZSM-5 catalysts do not show the formation of CO_2_ which implied that the competitive water-gas shift reaction (WGS: CO + H_2_O → CO_2_ + H_2_) which is typical of very poorly reduced Co-based FTS catalysts does not take place, or at least negligible. Few other reports^12, 13^ also did not observe the formation CO_2_ in the products distribution and authors ascribed this to high selectivity towards CH_4_ formation due to the presence of either unreduced cobalt species or to small cobalt particles strongly interacting with the support. However, Martínez *et al*.^20^ reported a general parallelism between the selectivity to CH_4_ and CO_2_ in a study on the influence of Co loading over mesoporous SBA-15, and they concluded that the higher CH_4_ selectivity displayed by well-dispersed low-reducible catalysts could be due, at least in part, to a higher extent of the WGS reaction occurring on unreduced Co species. Both Co/ZSM-5 and CoOx/ZSM-5 catalysts showed the formation of iso-paraffin (i-C_4_H_10_), however, the ratio of iso/n paraffin is relatively higher for CoOx/ZSM-5 obviously due to increased acidity (Fig. 8) and enhanced textural properties (Fig. 2 and Table 1) as a result of the OxA functionalization. Studies^21, 26, 31, 34^ have shown that increased acidity and porosity in zeolite supported catalysts are favorable for n-paraffin skeletal isomerization.

#### Hydrodeoxyhenation (HDO) of oleic acid

**Figure 11:**
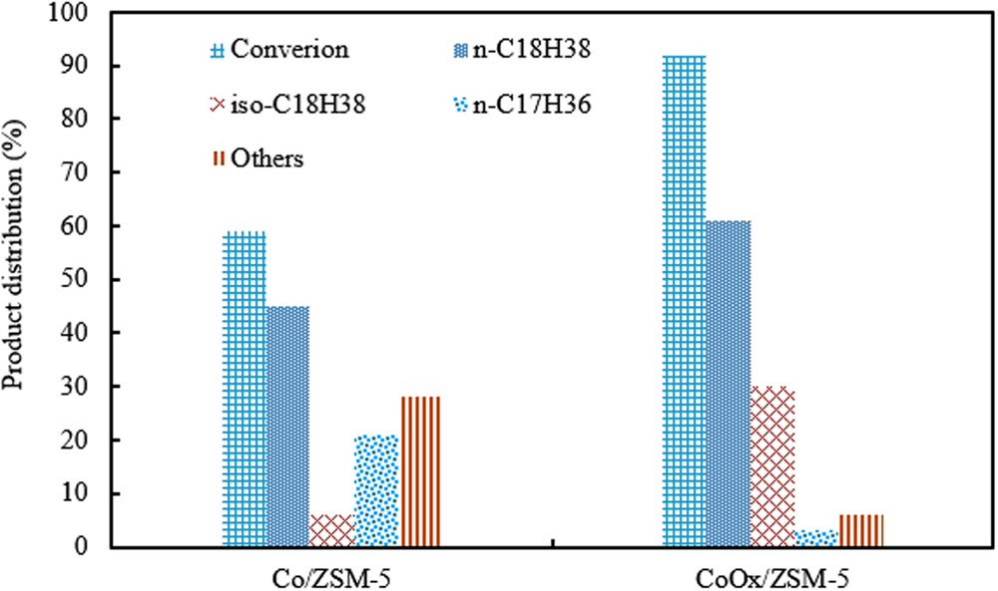
Conversion of oleic acid and product distribution over Co/ZSM-5 and CoOx/ZSM-5 catalysts. Reaction conditions: T = 360 °C, hydrogen pressure = 20 bar, agitator speed = 2000 rpm, reaction time = 1 h and catalyst loading 20 mg. Others = stearic acid, octadecanol and octadecene.

In order to further evaluate and appreciate the influence of the OxA functionalization, both Co/ZSM-5 and CoOx/ZSM-5 catalysts were tested for the HDO of oleic acid (OA) at previously established best observed reaction conditions of 360 °C and 20 bar^31, 34^. The result is shown in Fig. 11 with OA conversions of 59% and 92% over Co/ZSM-5 and CoOx/ZSM-5, respectively. The comparably higher OA conversions on CoOx/ZSM-5 was ascribed to the lower Co particle sizes with higher dispersion and enhanced reducibility as seen in the characterization results. The comparably lower Co species particle sizes according to the HRTEM result increased the number of available Co species particles in CoOx/ZSM-5, and this is supported by its higher metal dispersion. Similarly, CoOx/ZSM-5 high and readily reducible Co metals guarantee the availability of higher number of active site for the HDO reaction. The products distribution over both catalysts are somewhat identical although the amounts drastically varies with CoOx/ZSM-5 showing superior HDO and isomerization activities compared to Co/ZSM-5. The superior HDO activity is directly linked to the combined high Co dispersion and reducibility as earlier mentioned, while the enhanced isomerization activity is tied to the high density of acid sites. Previous studies^31, 34^ have shown that the acid sites of supported catalyst are favorable for the skeletal isomerization of hydrodeoxygenated n-paraffin, and the best isomerization temperature in those studies was 360 °C. Furthermore those studies showed that the production of considerable amount of isoparaffin had a favourable effect on the cold flow properties (e.g. cold filter plugging point), because the freezing point of a typical isoparaffins is significantly lower than that of its corresponding n-paraffins^34^. In simple terms, this implied that the temperature at which a given n-paraffin began to freeze, the corresponding isoparaffin is still essentially in liquid state.

**Figure 12:**
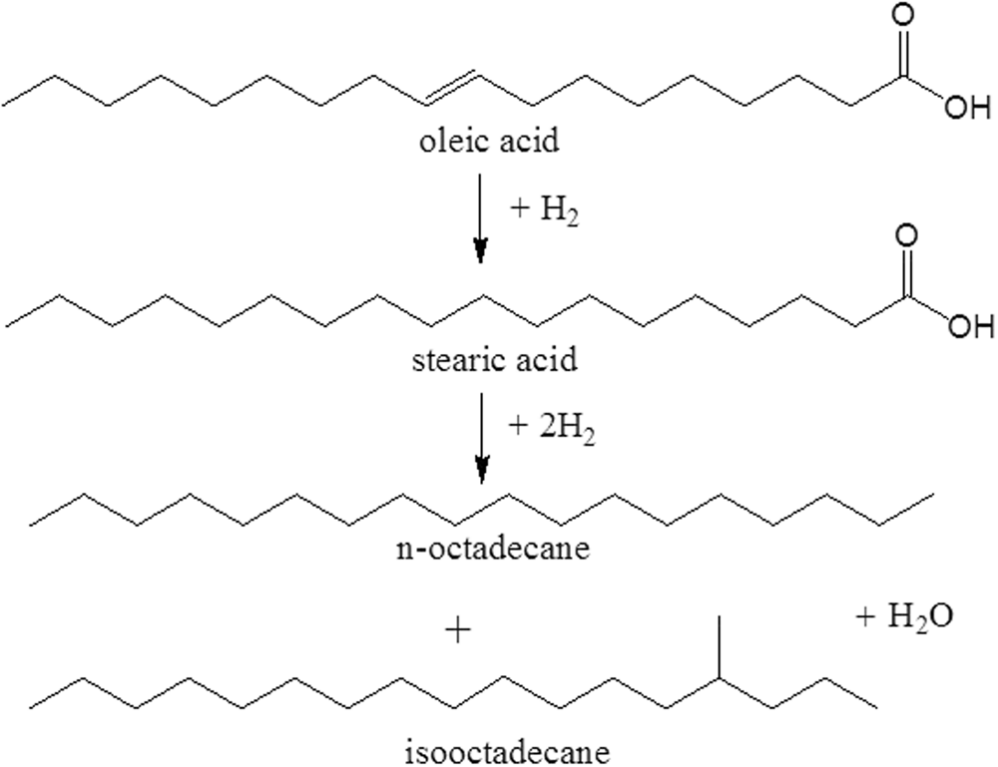
Schematic of HDO of oleic acid to paraffin biofuel via stearic acid.

Both catalysts showed the formation of C_17_H_36_ which implied that there are instances of decarboxylation competing with HDO process through which part of the oxygen in the OA functional group was removed as CO_2_. Accordingly, the amount of CO_2_ formed was 0.07 and 0.4 mmol CO_2_/mmol OA for CoOx/ZSM-5 and Co/ZSM-5, respectively. The formation of CO_2_ indicated that there is loss of one carbon atom leading to decrease in the energy density. Therefore since the decarboxylation process is more pronounced over Co/ZSM-5, the OxA functionalization is effective towards achieving high energy density biofuel with exceptional cold flow properties. There are other species that were observed in the products such as stearic acid, octadecanol and octadecene with stearic acid amounting to over 90% and 94%, respectively over Co/ZSM-5 and CoOx/ZSM-5, and they are lumped up and referred to as “*Others*”. According to previous reports^26, 31, 35^, the catalytic HDO of OA proceeds in two stages, firstly the saturation of the C = C, followed by the removal of the O_2_ as water (Fig. 12), thus, this explains the why stearic acid was present among the products. Similarly, the presence of octadecanol and octadecene were due to certain parallel reaction where stearic acid was reduced to octadecanol which was subsequently dehydrated to octadecene^36^. Based on the result shown in Fig. 11, it is clear that the OxA functionalization was also effective in enhancing selectivity to the desired product by minimizing the extent of competing reactions.

## Conclusion

The influence of OxA functionalization on ZSM-5 supported Co catalyst has been studied in this report. The characterization result showed that the functionalized cobalt oxalate supported ZSM-5 catalyst (CoOx/ZSM-5) has superior textural properties, higher metal dispersion, reduced and readily reducible Co species compared to the conventiaonal cobalt supported on ZSM-5 catalyst (Co/ZSM-5). The activites of both Co/ZSM-5 and CoOx/ZSM-5 were evaluated in both Fischer-Tropsch synthesis and hyrdodeoxygenation (HDO) of oleic acid (OA) in paraffin biofuel. The Time-on-stream (TOS) evolution of CO conversion over Co/ZSM-5 and CoOx/ZSM-5 catalysts were 74.28% and 94.23% at 0.5 h TOS and their selectivity to C_5+_ HC production were 63.15% and 75.4%, respectively at reaction conditions of 240 °C, 2.0 MPa, H_2_/CO = 2, GHSV = 1000 h^−1^ at 0.3 g catalyst loading. Similarly, in the HDO process, the OA conversion were 59% and 92% over Co/ZSM-5 and CoOx/ZSM-5, respectively. In addition CoOx/ZSM-5 showed superior HDO and isomerization activities compared to Co/ZSM-5. Thus, the OxA functionalization of Co is expedient.

## Electronic supplementary material

Supplementary information
