# Supplementary material for: Influence of oxalate ligand functionalization on Co/ZSM-5 activity in Fischer Tropsch synthesis and hydrodeoxygenation of oleic acid into hydrocarbon fuels
Source: Sci Rep. 2017 Aug 30;7:10008. doi: 10.1038/s41598-017-09706-z (PMC5577191; doi:10.1038/s41598-017-09706-z)
Supplement: Supplementary file 1 — Supplementary information [file 41598_2017_9706_MOESM1_ESM.doc]

**Influence of oxalate ligand functionalization on Co/ZSM-5 activity in Fischer Tropsch synthesis and hydrodeoxygenation of oleic acid into hydrocarbon fuels**

Olumide Bolarinwa AYODELE

Department of Chemical Engineering, Universiti Teknologi PETRONAS, 32610 Bandar Seri Iskandar, Perak, Malaysia.

E-mail: [ayodele_olumide@yahoo.com](mailto:ayodele_olumide@yahoo.com); [ayodele.olumide@utp.edu.my](mailto:ayodele.olumide@utp.edu.my); Tel +60164955453

**Supplementary information**


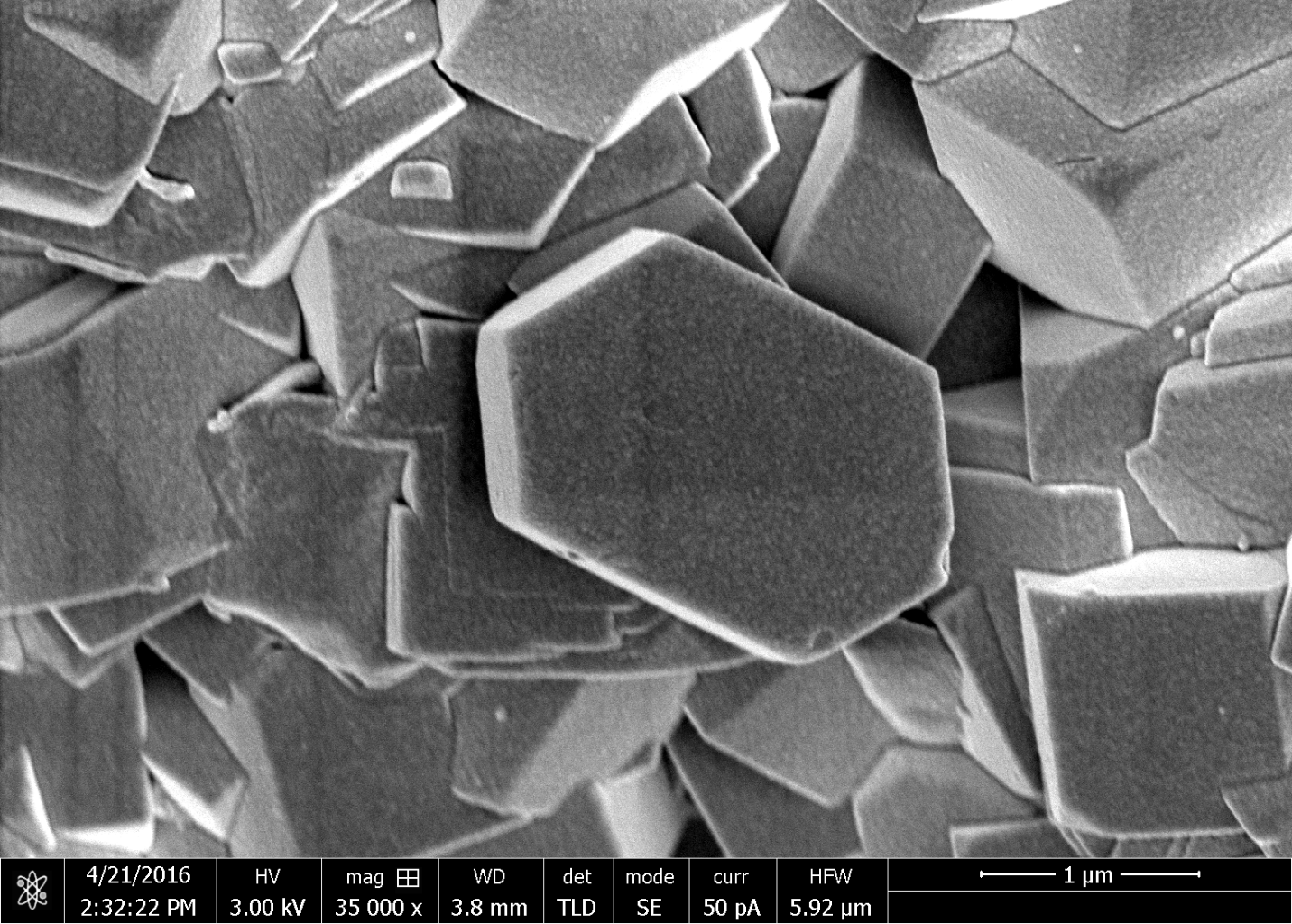


Figure S1. SEM image of ZSM-5 showing the average particle size to be 1.85 micrometer.
